# Using Virtual Scanning to Find Optimal Configuration of a 3D Scanner Turntable for Scanning of Mechanical Parts

**DOI:** 10.3390/s21165343

**Published:** 2021-08-07

**Authors:** Tomáš Kot, Zdenko Bobovský, Dominik Heczko, Aleš Vysocký, Ivan Virgala, Erik Prada

**Affiliations:** 1 Department of Robotics, Faculty of Mechanical Engineering, VSB-Technical University of Ostrava, 17. Listopadu 2172/15, 708 00 Ostrava-Poruba, Czech Republic; zdenko.bobovsky@vsb.cz (Z.B.); dominik.heczko@vsb.cz (D.H.); ales.vysocky@vsb.cz (A.V.); 2 Faculty of Mechanical Engineering, Technical University of Košice, 04200 Košice, Slovakia; ivan.virgala@tuke.sk (I.V.); erik.prada@tuke.sk (E.P.)

**Keywords:** 3D scanning, point cloud, time-of-flight, TOF, scanner, camera, simulation

## Abstract

The article describes a method of simulated 3D scanning of triangle meshes based on ray casting which is used to find the optimal configuration of a real 3D scanner turntable. The configuration include the number of scanners, their elevation above the rotary table and the number of required rotation steps. The evaluation is based on the percentage of the part surface covered by the resulting point cloud, which determines the ability to capture all details of the shape. Principal component analysis is used as a secondary criterion to also evaluate the ability to capture the overall general proportions of the model.

## 1. Introduction

3D scanning is the process typically used to get numerical data representing the shape of a real physical object. The subsequent use of such data depends on the specific project—some common uses include visualization [1], mapping [2], reverse engineering and rapid prototyping [3], quality control [4], prosthetic [5], digitization of important objects [6], gesture control, autonomous navigation in unknown terrain [7,8], etc.

There are a variety of commonly used technologies for 3D scanning. The first category includes contact (tactile) systems that are typically used in metrology [9], as acquiring a full 3D scan of a complex shape using this technology is time-consuming. However, the advantages of tactile systems are accuracy and insensitivity to optical properties of the scanned surfaces. Non-contact active technologies include two basic groups of technologies—time of flight and triangulation. Time-of-flight scanners use laser rays to progressively probe the scanned surface [10], whereas time-of-flight cameras are able to provide a full 2D image with a depth component in a single operation [11] usually using a built-in shutter (range-gated imagers [12]) or modulated light [13]. Triangulation-based 3D scanners use a combination of a laser emitter that emits laser points or strips and a detector located in offset positions [14]. This technique is also used by structured-light 3D scanners that project multiple stripes or other complex patterns at once [15]. Non-contact passive systems do not emit any kind of radiation and usually detect reflected visible or infrared light. Stereoscopic systems use two cameras separated by some horizontal distance and determine depth of individual pixels by comparing the two images [16], while photometric systems use a single camera that takes images at varying lighting conditions [17]. Photogrammetry reconstructs the 3D shape of an object by processing photographic images taken from multiple positions [18].

In many of the applications mentioned above, it is necessary to process the data, sometimes even in real-time, by some specific algorithm or a neural network [19]. To properly configure, verify and debug these algorithms, or teach the neural network, a huge set of testing data may be required—which may be hard or even impossible to get using actual 3D scanning of real objects. Therefore, virtual or simulated 3D scanning can be used instead, where the functions and properties of a 3D scanner are simulated in software and the scanned object is a 3D model of the object created in a CAD system. The absence of physical objects and scanners make the process easier, much faster and cheaper.

When a full 3D scan of an object is required, it is necessary to scan the object from multiple angles and combine the scans together, because the surfaces facing away from the scanner can never be detected by it. For concave objects, there is another common problem where a part of the object can block visibility of other parts of the same object by casting a shadow on it. This is another field where virtual scanning can be used in order to find the optimal configuration and number of scans required to obtain a scan covering a sufficient portion of the object surface area.

Although 3D scanning and point cloud processing is a very popular and trending topic, applications of virtual (simulated) scanning in particular are not mentioned very often. Some previous work in this area include, for example, the simulated hand-held scanner in virtual reality for the Oculus Rift head-mounted display [20], where the authors created a full simulation of the behavior of a hand-held scanner which allows easy testing, training or verification of the process of creating point clouds of CAD models without the presence of physical scanners or the scanned objects.

Another study focused on development of a new laser detection and ranging (LADAR) simulator created in MATLAB [21] with the focus mainly on accuracy of the simulation by properly simulating the important aspects of the laser beam while maintaining a good calculation speed. The intended target audience of this simulator are developers of LADAR systems who need to devise and configure their data processing algorithms in an effective way. Similar simulators are described in [22] with a mathematical model that models waveforms using a novel hexagonal sampling process applied across the LADAR beam footprint; in [23] where the authors used a focal plane array with Geiger mode detection; in [24] where the main focus is on reduction of the calculation time by using an optimized geometric model and an incremental algorithm with parallel processing based on a CUDA (Compute Unified Device Architecture) enabled GPU (Graphics Processing Unit).

The authors of [25] used simulated scanning to determine the ideal orientation angle of a LADAR system for the purpose of autonomous navigation and mapping, by assessing the density, coverage and accuracy or the created 3D maps. The simulations are performed in the Blender tool.

Complex simulation systems such as CoppeliaSim (previously known as V-Rep) provide tools for simulation of simple sensors based on ray casting. In these systems, simulated sensors are typically used as a component of the virtual scene built for verification of some navigation or control algorithm in mobile [26] or industrial [27] robotics. Similarly, ROS (Robot Operating System)—a system commonly used not only to control real robots but also to perform simulations—contains packages for simulation of laser scanners [28]. Evidently, simulation of laser scanners is not a new idea, but it can be used in new types of applications and with new goals.

### Featured Application

This article considers a specific application where the 3D scanning of a real mechanical part is done in order to analyze and recognize the part by comparing the scan with the database of all the parts of some complex machine. The scanning device used in this particular use case is in the form of a turntable with one of more 3D scanners placed in different elevations. The user places an unknown mechanical component on the table. Then, the system should scan it and return the part number or name of the component. The scanning device must be able to properly scan objects of various shapes, including concave, with the maximal dimensions of 400 mm × 400 mm × 400 mm.

The algorithm applied for shape recognition is not a topic of this paper, although it is undoubtedly a very interesting and trending area with focus shifting to deep learning methods nowadays [29]. The algorithm used in our case is based on a combination of principal component analysis [30] and the RANSAC algorithm for primitives decomposition [31]. The algorithm is described in detail and verified in [32].

The aim of this paper is to propose a method of finding the recommended configuration of the scanning device before building the real one. The optimal configuration could be discovered analytically for a specific scanned object or a limited set of similarly-shaped objects, but this is impossible for shapes that are not known in advance as there are no data to base the algorithm on. Instead, virtual scanning is used on a large set of objects of different sizes and shapes and the idea is to use statistics to select the optimal configuration. Therefore, the selected configuration represents a compromise that should provide the best possible scanning results for a variety of mechanical parts without the need of frequent adjustments.

This goal differs from the topics related to virtual scanning solved by other researchers (see the previous section), where the main focus is either high accuracy of scanning or application in the field of robot control and navigation.

In the following text, the term scanner represents the physical sensor (or its simulated counterpart) in a specific position in space, while scan represents the point cloud acquired by performing one scanning operation of a scanner in one position, unless explicitly specified that merged scans from multiple scanner positions are meant instead.

## 2. Simulated Scanning

The proposed virtual scanner simulates the behavior of a time-of-flight (TOF) scanner, TOF camera [33] or a triangulation scanner [14]. The simulation is programmed in C++ and uses Direct3D 11.0 [34] for 3D graphics rendering and the DirectXMath library [35] for vector algebra calculations. This custom solution provides better performance than simulation systems such as CoppeliaSim and allows easy modifications of the scanner algorithm and simulation of principles like triangulation or scanning errors.

### 2.1. Simulating Time-of-Flight Sensors

This type of scanner uses a laser ray (beam) to probe the scanned surface and measures the time required for the light to reach the surface, reflect and return back to the sensor in the scanner. As the speed of light is known, the measured time can be directly used to calculate the distance. One point on the surface is measured at a time, therefore performing a 2D or 3D scanning requires change of the laser ray direction in one or two axes, which is typically done by rotating mirrors.

Getting a whole set of distance measurements without a tilting laser beam is possible using a time-of-flight camera [33], which emits a wide flash of light and measures the return times separately—but virtually at the same time—for all pixels of the CMOS sensor.

To simulate the behavior of a time-of-flight 3D scanner with an acceptable adherence to reality, mathematical models of the following phenomena should be made:

- measuring one distance by casting a ray of light and tracking it back to the sensor after bouncing off the surface;
- changing the direction of the ray in two axes by defined step angles, within defined boundaries;
- accuracy and noise.

As far as the distance measuring is concerned, it is not necessary to simulate the laser beam propagation through the atmosphere and its reflection on the surface. The simulation is based on casting a 3D vector ray from the location of the scanner in the proper direction, finding the intersection of the ray with the scanned surface and calculating the distance between the intersection point and the origin of the ray. The change of ray direction is also very easy to simulate by vector algebra.

Although time-of-flight cameras do not use laser beams to probe the surface, the resulting effect is very similar (the distances to individual pixels in the image are measured), so it is possible to simulate also TOF cameras the same way—the depth value for every pixel of the CMOS sensor is acquired in the simulation by casting a separate ray.

#### 2.1.1. Calculating the Ray Distance

**Figure 1:**
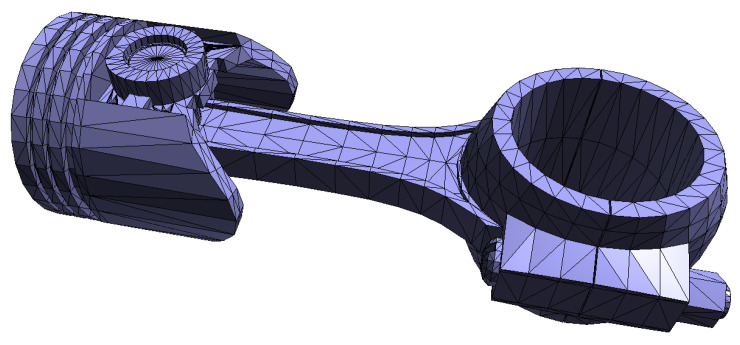
An example of a triangle mesh.

One of the common ways of representing generic complex shapes in computer graphics is by triangle meshes. The surface of the object is approximated by a set of triangles, where every triangle shares each of its three sides with other adjacent triangles, as can be seen in the example in Figure 1.

**Figure 2:**
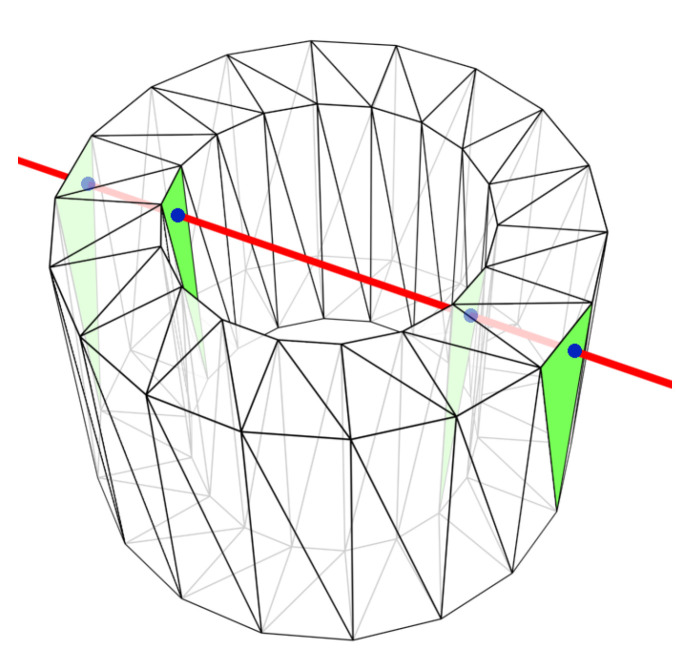
A ray intersecting four triangles of a concave mesh.

When probing a closed triangle mesh with a ray of a known origin and direction, the ray will always intersect an even number of triangles—at least two (one front-facing and one rear-facing). For concave shapes, the number of intersection can be higher, but pairs of a front-facing and a rear-facing triangle are always formed (Figure 2). The first point of contact (the shortest distance) is the important value, because in reality the ray is stopped and reflected when it hits the surface for the first time. The mathematical model works in a different way and all triangles must be tested, unless the triangles are always sorted by their distance from the ray origin–which would be unacceptably time-consuming.

**Figure 3:**
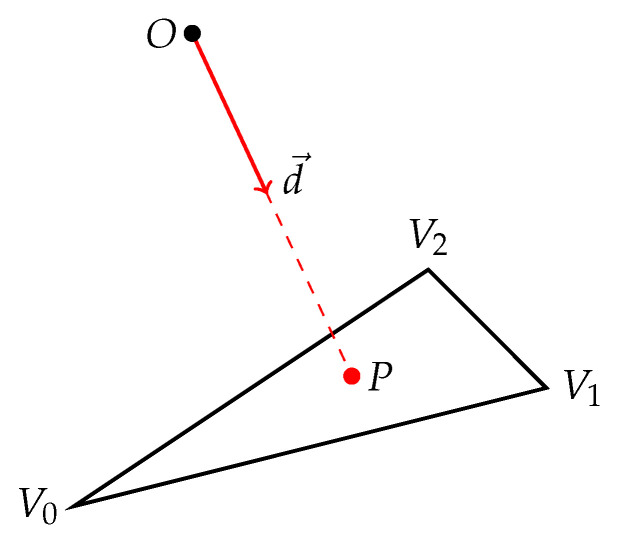
Ray-triangle intersection.

Rear-facing triangles intersected by the ray as it leaves the inner volume of the object can be discarded quickly to save some calculation time. For this, the normal vector ${\vec{n}}_{i}$ of each mesh triangle should be pre-calculated and stored in advance

(1) $$\vec{n}=\left(V_{1}-V_{0}\right)\times \left(V_{2}-V_{0}\right),$$

where $V_{0}$, $V_{1}$ and $V_{2}$ are the triangle vertices (Figure 3). It is not necessary to normalize the vector $\vec{n}$, as only its direction is important, not the length. Rear-facing triangles are then detected simply by calculating the dot product between the normal vector $\vec{n}$ and the ray direction vector $\vec{d}$ (Figure 3)

(2) $$a=\vec{d}\cdot \vec{n}$$

and checking its sign; if a≥0, then the triangle is not front-facing and can be skipped for further processing.

The intersection of a ray and a triangle is then found using the fast algorithm proposed by Möller and Trumbore [36]. The algorithm takes as input the ray origin *O*, ray direction $\vec{d}$ and the vertices $V_{0}$, $V_{1}$ and $V_{2}$ of the triangle and returns a boolean value indicating whether an intersection has been found, together with the *h* value representing the distance from *O* to the point of contact *P* (Figure 3).

Rough approximation of measurement error and noise in the data can be included by multiplying the distance *h* by a random value within the range corresponding to the declared accuracy of the real sensor *e* in percents

(3) $$h^{\prime }=h\cdot \mathrm{rand}\left(1-\frac{e}{100},\;1+\frac{e}{100}\right).$$

Finally, the position of the intersection point *P* (including distance error noise) can be calculated as

(4) $$P=O+\vec{d}\cdot h^{\prime }.$$

The ray direction $\vec{d}$ could also be randomly altered by a small amount, especially when simulating a TOF scanner based on a laser beam. However, this is not included in our simulation. The algorithm for finding the first point of contact of a single ray with the triangle mesh is described by pseudo-code in Algorithm 1.
**Algorithm 1:** Finding the first point of contact of a single ray with the whole mesh
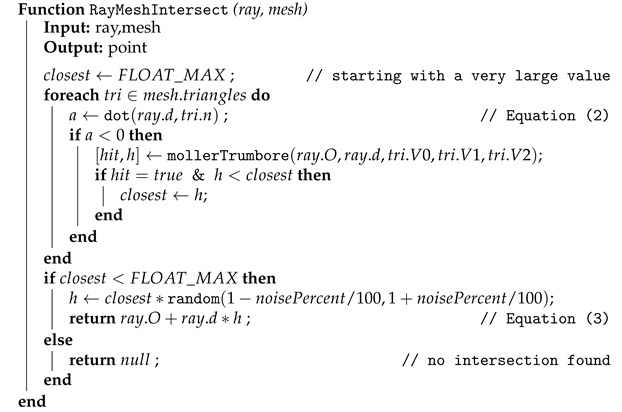


#### 2.1.2. Acquiring the Simulated Point Cloud

**Figure 4:**
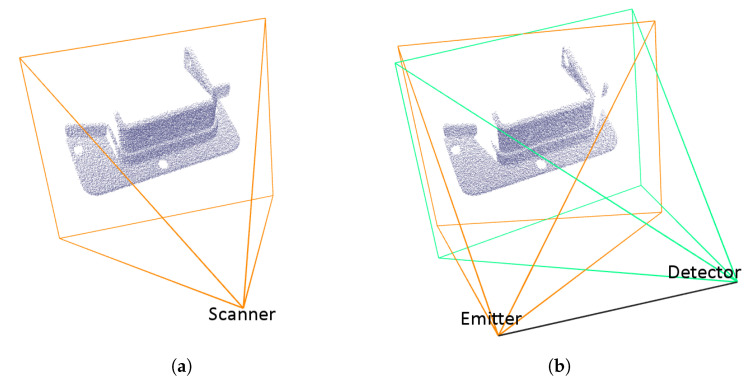
A sample point cloud acquired by simulated 3D scanning and pyramids representing the view volumes of the simulated scanners: (**a**) a TOF scanner; (**b**) a triangulation scanner.

**Figure 5:**
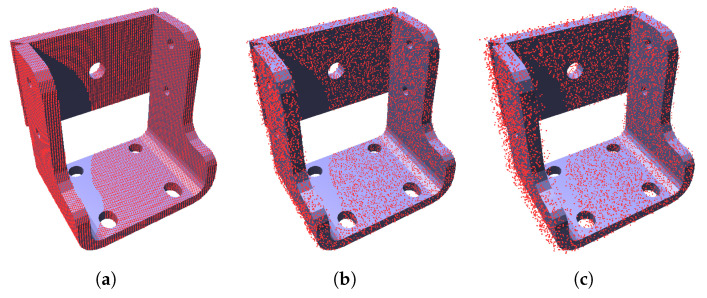
Scan made by a single virtual scanner with various amount of simulated noise: (**a**) no noise; (**b**) low amount of noise (1 % of distance measurements); (**c**) high amount of noise (3 % of distance measurements).

Every ray that hits the mesh creates one point *P* of the point cloud at the closest intersected front-facing triangle, as described in Algorithm 1. The whole scan in the form of a point cloud is acquired by making the appropriate number of ray tests according to the parameters of the TOF scanner or camera (Figure 4a). For a TOF camera, the number of cast rays depend on the resolution of the CMOS sensor, as the depth of every pixel is simulated by casting one ray. For a TOF scanner, the number of cast rays directly corresponds to the angle of view and step angle of the laser. The whole algorithm for acquiring the simulated scan (point cloud) for a single scanner is described by pseudo-code in Algorithm 2. An example of a simulated single scan of a model with various amount of noise in shown in Figure 5.
**Algorithm 2:** Acquiring the whole simulated scan for one TOF scanner
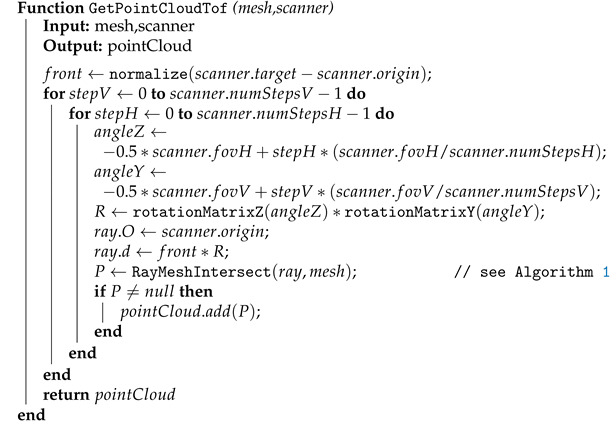


### 2.2. Simulating Triangulation Scanners

Although triangulation scanners work in a different way than TOF scanners, it is not necessary to simulate the process in full detail. The biggest difference that must be included in the simulation is the fact that a point on the surface can be added to the point cloud only if it is in the direct field of view of the emitter and the detector at the same time. This lowers the surface coverage achievable by a single scan, because more areas are in shadow.

The triangulation depth calculation does not have to be simulated; the depth can be measured in the same way as for the simulated TOF sensors instead. Properly calculating the position and distance of the scanned point by triangulation based on the angle of the emitted laser ray and the angle at which the intersection point of this ray with the surface is visible by the detector camera would give the same geometric results at the cost of additional computation power. Thus, the virtual laser ray is cast from the emitter position (see Figure 4b) and the intersection with a mesh triangle is found as for a TOF scanner. However, an additional important step is added—another ray is cast from the detector position towards the intersection point and the point is discarded if this second ray hits another triangle of the mesh before reaching the target point. This imaginary second ray does not represent a laser ray emitted by the real scanner; its purpose is merely to check direct line of sight from the detector. The practical impact on surface coverage caused by occlusions can be seen by comparing Examples (a) and (b) in Figure 4. The modified version of Algorithm 2 is shown in Algorithm 3 (the code is simplified to show the principle; a better handling of the distance noise is required in this case).
**Algorithm 3:** Acquiring the whole simulated scan for one triangulation scanner
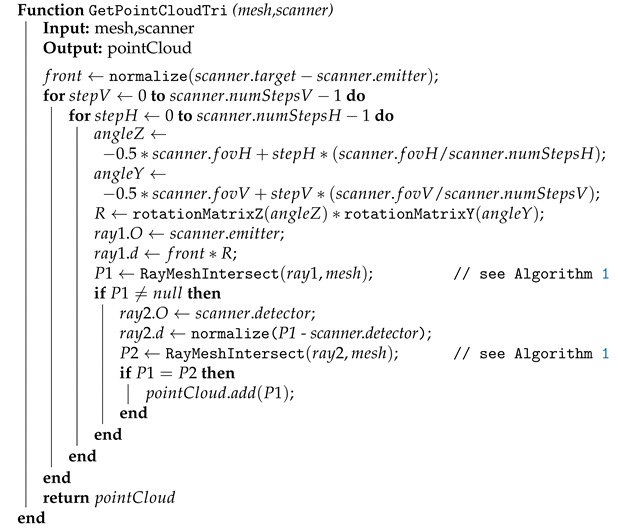


### 2.3. Simulating a Scanning System with a Turntable

To scan an object from multiple angles while using only one physical scanner, a rotary table (turntable) is commonly used [37,38]. The object rotates on the table in discrete steps and the scanner or camera placed above the table creates individual point clouds that are then finally merged into the resulting cloud.

**Figure 6:**
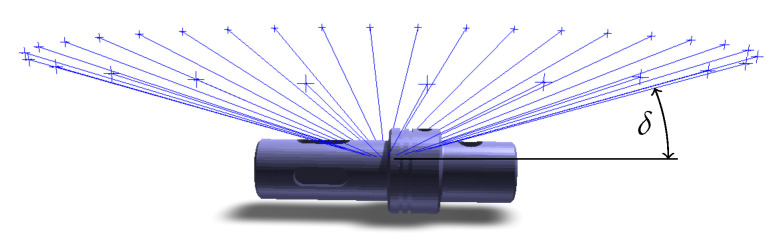
Positions (blue crosses) and orientations (blue lines) of scanners rotated around the fixed turntable (30 steps). δ represents the elevation angle.

For simulated scanning, it is easier to calculate a new position and orientation of a virtual scanner than to rotate the potentially very complex triangle mesh. Therefore, the table is considered fixed and the scanner rotates around it instead. An example of scanner positions for elevation angle $\delta =20^{\circ }$ and 30 rotating steps is shown on Figure 6.

Especially in the case of a large number of steps, the individual point clouds overlap by a big factor. This would excessively increase the number of points in the point cloud without adding too much additional useful information. To reduce the complexity of the point cloud, remove duplicated points and achieve better uniform distribution of points, the final point cloud is filtered by voxelization [39]. The whole algorithm for a TOF scanner is described by pseudo-code in Algorithm 4; the version for a triangulation scanner would be slightly extended to calculate the locations of the emitter and detector instead of just one location for each scanner.
**Algorithm 4:** Acquiring the merged scan for multiple rotations of a turntable
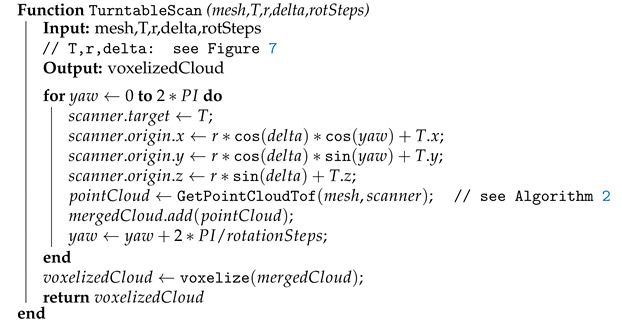


## 3. Simulation Setup

The TOF scanner(s) used in the simulations have the following parameters:

- horizontal angle of view $55^{\circ }$;
- vertical angle of view $40^{\circ }$;
- resolution 640×480;
- 0.5% of noise.

**Figure 7:**
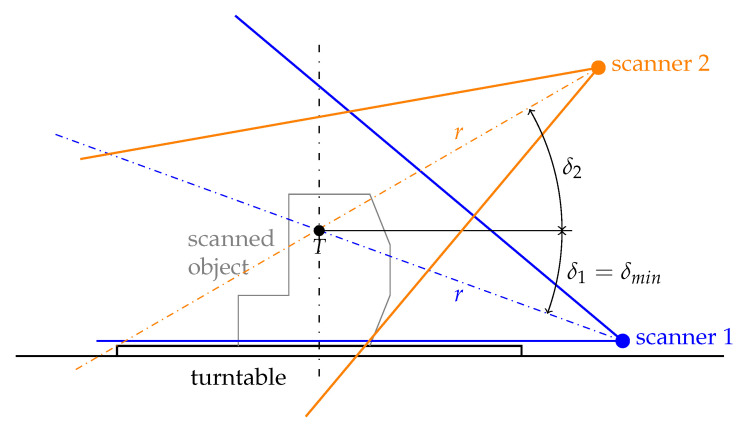
Side view of the turntable with two example locations and horizontal viewing angles of scanners (elevation angles $\delta_{1}$ and $\delta_{2}$, in this case $\delta_{1}=\delta_{min}$).

The turntable setup is shown on Figure 7; the scanners are always pointing towards the point *T* located 200 mm above the table (half of the given maximal dimension of the objects) and are 590 mm far from this point. The minimal elevation angle is $\delta_{min}=-20^{\circ }$ and represents for this configuration the best possible placement of a scanner for scanning the bottom parts of the objects.

### 3.1. Tested 3D Model Samples

**Figure 8:**
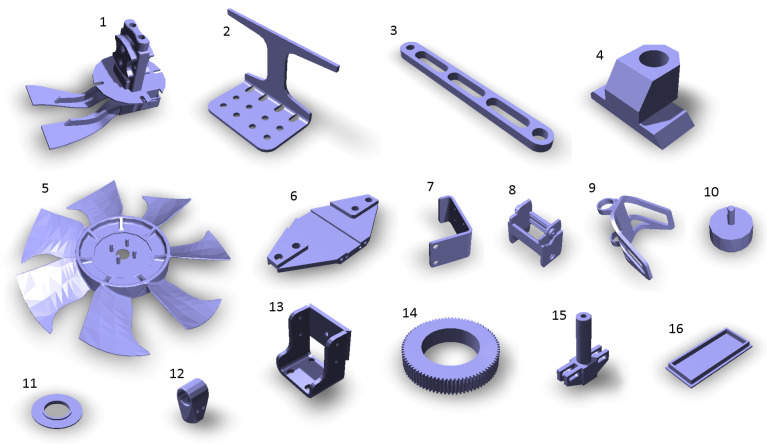
The 16 selected sample models from the total number of 200 models used in the simulations.

The experiments were executed for 200 STL (stereolithography) [40] models of real mechanical parts of various shapes and sizes—including shafts, flanges, covers, housings, gears, levers, etc. Figure 8 shows 16 samples as a demonstration of the shape variety. This figure also indicates the chosen orientation of the objects when placed on the virtual scanning turntable; other orientations would lead to different results. The orientations were chosen manually for each model with the following conditions (it is of course not always possible to satisfy all of them):

- The most features of the shape point upwards.
- The bottom surface is the flat-most surface of the model,.
- The orientation must be physically achievable in reality (static stability and also stability during the turntable rotation process).

### 3.2. Evaluation Criterion

Based on the particular use case where the final point cloud is used to identify an unknown mechanical part by comparing the point cloud with a database of models, the chosen criterion for evaluation of the final point cloud is the percentage of the part surface covered by the points. This choice is based on the assumption that, to successfully recognize similar parts that differ only in some details, it is necessary to have all the details represented in the point cloud.

The surface coverage could be calculated by comparing the point cloud acquired by scanning with the calculated area of the model surface. As mentioned above, the point cloud is voxelized after scanning, so it would be possible to approximate the area covered by the points using the known voxel size—the area would correspond to the number of points multiplied by the square of the voxel size. However, this approach causes some inaccuracy for model surfaces that are not parallel to the voxel sides, for example in the case of a 45° angle between the surface normal and the voxel orientation, the error factor would be $\sqrt{2}$.

**Figure 9:**
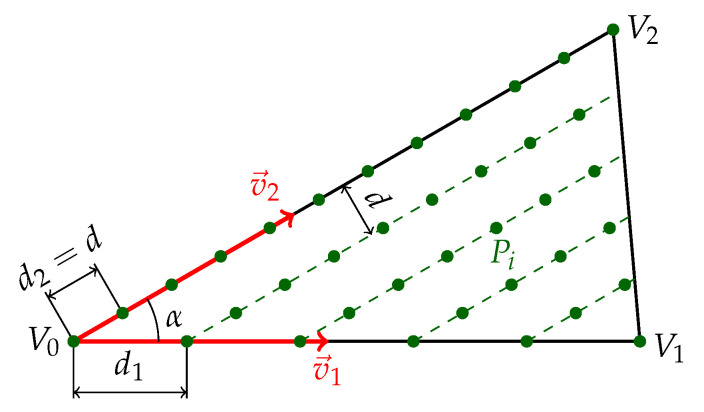
A method of semi-even distribution of points on a single triangle.

Thus, a different method was chosen: the surface coverage is calculated by comparing the point cloud acquired by scanning with a reference point cloud covering semi-evenly the whole surface. This reference point cloud cannot be created naively by making a point in every vertex of the triangle mesh, because large planar faces are usually represented by a small number of large triangles with big distances between vertices. The following simple method of semi-even distribution of points over a triangle mesh was thus proposed (see also Figure 9).

For each triangle of the mesh, let us denote the vertices $V_{0}$, $V_{1}$ and $V_{2}$. Two normalized side vectors can be calculated as follows:

(5) $$\begin{array}{cc} {\vec{v}}_{1} & =\frac{V_{1}-V_{0}}{|V_{1}V_{0}|}, \end{array}$$

(6) $$\begin{array}{cc} {\vec{v}}_{2} & =\frac{V_{2}-V_{0}}{|V_{2}V_{0}|} \end{array}$$

and the angle between those vectors is

(7) $$\alpha =acos({\vec{v}}_{1}\cdot {\vec{v}}_{2}).$$

Positions of all points covering the triangle are then given by the following equation:

(8) $$P_{i}=V_{0}+{\vec{v}}_{1}d_{1}x+{\vec{v}}_{2}d_{2}y,$$

where *d* is the chosen pitch of points, $d_{2}=d$, $d_{1}=\frac{d}{sin(\alpha )}$ and variables *x* and *y* successively gain integer values from 0 until the $A_{1}A_{2}$ triangle side is reached, which is given by the following conditions:

(9) $$\begin{array}{ll} 0\leq d_{1}x\;<|V_{0}V_{1}|, & \;\;\;\;\;\;\;\;\;\;\;\;\;\;\;\;\;\;\;\;\;\;\;\;\;\;\;\;\;\;x\in Z^{+} \end{array}$$

(10) $$\begin{array}{ll} 0\leq d_{2}y\;<|V_{0}V_{2}|\left(1-\frac{d_{1}x}{|V_{0}V_{1}|}\right). & \;\;\;\;\;\;\;\;\;\;y\in Z^{+} \end{array}$$

**Figure 10:**
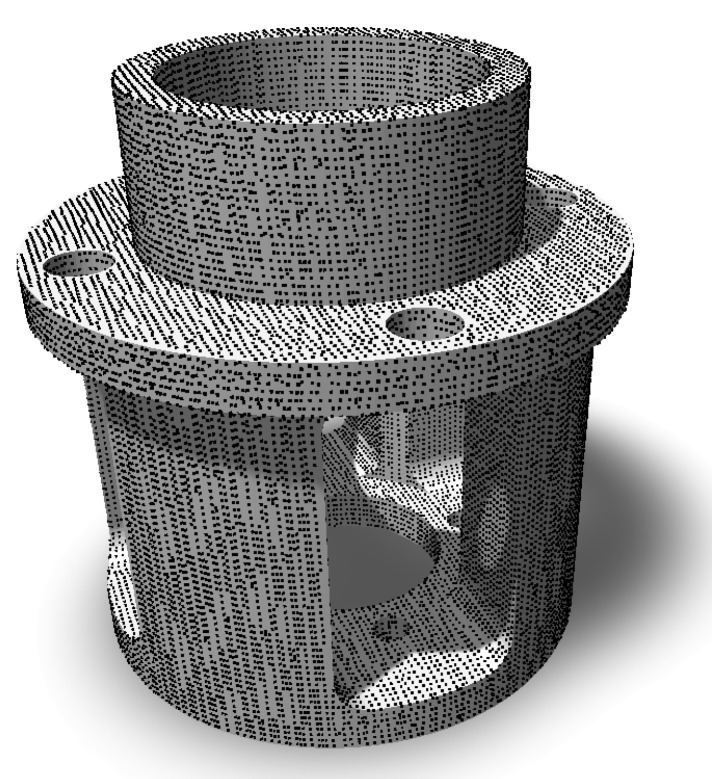
An example of a reference point cloud covering evenly the whole surface of a model.

Due to the individual coverage of separate triangles, the constant and even pitch of points is not preserved on triangle borders. This is dealt with by performing voxelization of the reference point cloud consisting of points covering all triangles with the same voxel size as for voxelization of the point cloud from scanning. This allows dividing the number of points in the scanned point cloud by the number of points in the reference point cloud and getting the approximate percentage of surface coverage. An example of a reference point cloud is shown on Figure 10.

## 4. Simulation Results and Discussion

Several separate experiments were performed with the goal of finding the optimal elevation of a single scanner, optimal combination of elevations of two scanners and the optimal number of turntable steps. The experiments were done using the simulation setup described above; the results are displayed primarily using the box plot diagram, where the height of each shaded rectangle represents the interquartile range (third quartile minus first quartile), which means that 50% of all values lie inside the rectangle. The horizontal line inside the rectangle represents the median, the cross represents the arithmetic mean and the small circles in each column represent the outliers.

All experiments were executed with the same amount of simulated random noise (0.5 % of the measured distance). As the impact of this type of simulated noise on surface coverage has uniform distribution, there is no need to make experiments for different noise levels.

### 4.1. Elevation of a Single Scanner

The first experiment tried to find the optimal elevation angle for a single scanner. The number of rotation steps of the turntable is 10 (increment angle 36°). The variable value in this experiment is the elevation angle δ with values in the interval $\delta \in \langle 0^{\circ },70^{\circ }\rangle$ and $10^{\circ }$ increments.

**Figure 11:**
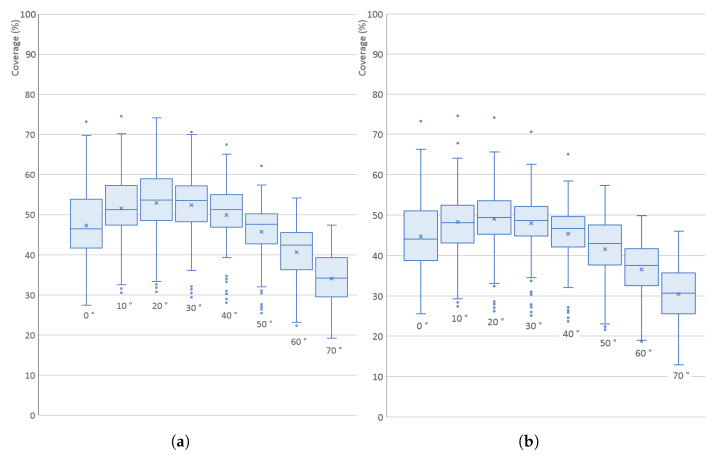
Surface coverage (%) for different elevation angles (°) of a single scanner: (**a**) simulated TOF scanner; (**b**) simulated triangulation scanner.

**Figure 12:**
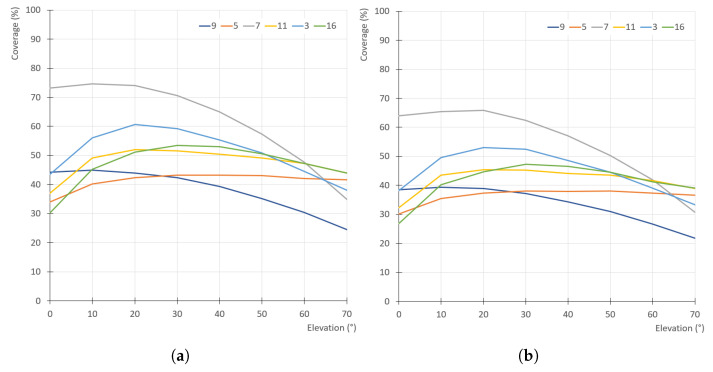
Surface coverage (%) for different elevation angles (°) of a single scanner for six selected concrete models in Figure 8: (**a**) simulated TOF scanner; (**b**) simulated triangulation scanner.

The results for all 200 models are shown in Figure 11 in the form of a box plot; the averaged, minimal and maximal values are also listed in Table 1. Figure 12 shows individual graphs for six selected models in Figure 8, namely Models 3, 5, 7, 9, 11 and 16.

**Figure 13:**
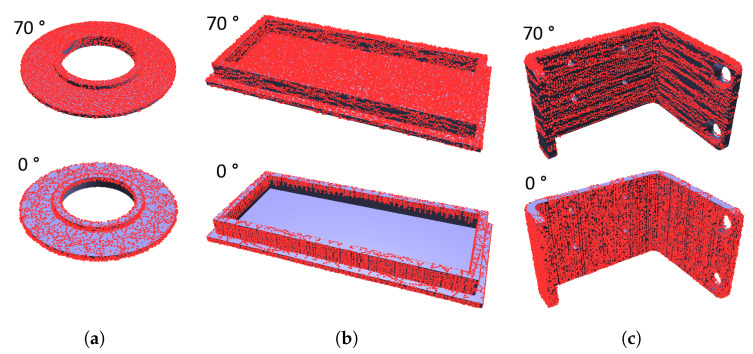
Comparison of point clouds acquired by simulated scanning using a single scanner at elevation $\delta =0^{\circ }$ and elevation $\delta =70^{\circ }$: (**a**) Model 11, better coverage for higher scanning angles; (**b**) Model 16, better coverage for higher scanning angles; (**c**) Model 7, better coverage for lower scanning angles.

The values have a significant spread, which is given by the different shapes of the models—for example, Models 11 and 16 (low flat circular or rectangular caps) have very small surface coverage when scanned from the side; Model 5 has a low percentage in the whole range (it has a large surface area oriented downwards); Model 7 has the highest percentage when scanned horizontally (see Figure 13). However, in Figure 11, it is quite clear that the optimal elevation is $\delta =20^{\circ }$, which has the highest arithmetic mean, median and also the third quartile.

Figure 11 and Figure 12 also show a comparison of the results for the TOF scanner used in all simulations with a triangulation scanner that has the same parameters, and the distance between its emitter and detector is *L* = 350 mm. The influence of elevation is almost exactly the same as for the TOF scanner, which is given by the fact that the triangulation scanner was oriented horizontally, and, although the separation of emitter and detector generally lowers the surface coverage (more areas of the object surface are in shadow), this is independent on the scanner elevation. As the difference is not significant, only the results for the TOF scanner are shown for the following simulations.

It is also obvious, and easily anticipated, that using only one scanner with a fixed elevation provides only small coverage of the whole surface—the maximum values are approximately 73% and the median is 54% (51% for the triangulation scanner). This number is also negatively affected by the low number of turntable rotation steps (10)—but, even when increased significantly, the coverage reaches only 60%. This is unacceptable for most uses, so multiple scanners (or multiple elevations of the same scanner) should be used.

### 4.2. Elevations of Two Scanners

This experiment tried to find the optimal elevation for a pair of scanners—which means either using two separate scanners mounted in fixed elevations or using one scanner that can automatically change its elevation during scanning.

As the idea is to scan the object from multiple different angles, the range of elevations was extended to comprise also the lowest possible angle $\delta_{min}=-20^{\circ }$ (see Figure 7) and the angles of both scanners always differ by at least $\delta_{2}-\delta_{1}=30^{\circ }$. Allowing one of the scanners to take the extreme lowest position enables at least some degree of coverage of the bottom parts and downward-oriented surfaces of the object. Elevations of both scanners were thus chosen according to the following conditions:

(11) $$\begin{array}{cc} \delta_{1} & \in \langle -20^{\circ },40^{\circ }\rangle , \end{array}$$

(12) $$\begin{array}{cc} \delta_{2} & \in \langle 10^{\circ },70^{\circ }\rangle , \end{array}$$

(13) $$\begin{array}{cc} \delta_{2} & \geq \delta_{1}+30^{\circ }. \end{array}$$

The angles change with the increment of $10^{\circ }$ and the number of rotation steps of the turntable is 10 (the same number as in the previous experiment).

**Figure 14:**
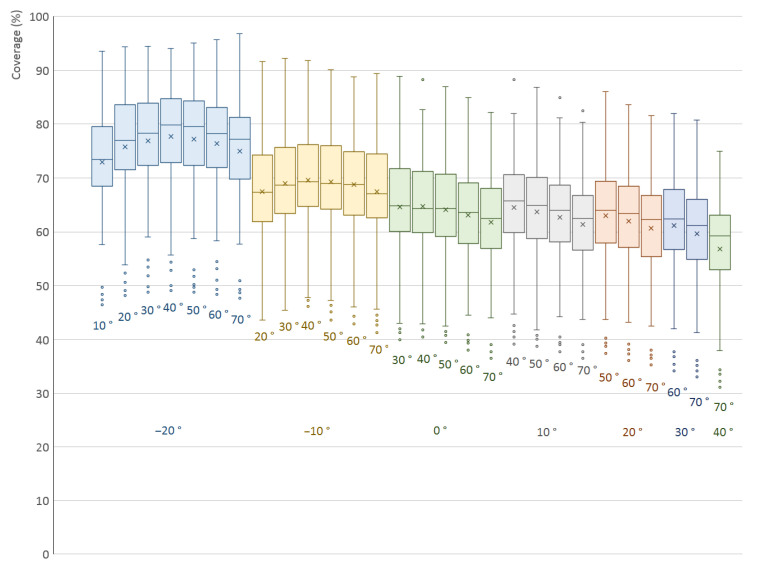
Surface coverage (%) for different elevation angles (°) of a two-scanner setup. Elevation angle $\delta_{1}$ of the first scanner is constant for each colored group of graphs and is listed at the bottom.

The results for all 200 models are shown in Figure 14 in the form of a box plot; the averaged, minimal and maximal values are also listed in Table 2. The spread of the values of different 3D models is very large, but the overall trend is clearly visible; the graphs show that the highest arithmetic mean, median and third quartile is for $\delta_{1}=-20^{\circ }$ and $\delta_{2}=40^{\circ }$. It is also worth mentioning that the graphs in the first group ($\delta_{1}=-20^{\circ }$) are higher than the other groups. This was expected, as this lowest position of the first scanner covers parts of the object that are not visible for a scanner located at the horizontal level or higher.

### 4.3. Number of Turntable Steps

The previous experiments were done with a fixed and relatively small number of rotation steps of the turntable (10). Increasing this number means performing more individual scans from different angles around the object in the horizontal plane, which should result in a point cloud that covers the surface better. This experiment was executed to verify this and also to find a potential upper limit on the number of steps above which further increasing has no additional positive effect.

This experiment was done for the optimal configuration of two scanners outlined in the previous section—elevations $\delta_{1}=-20^{\circ }$ and $\delta_{2}=40^{\circ }$. The number of steps is variable and ranges from 2 to 10 in steps of 2 and then from 20 to 70 in steps of 10.

**Figure 15:**
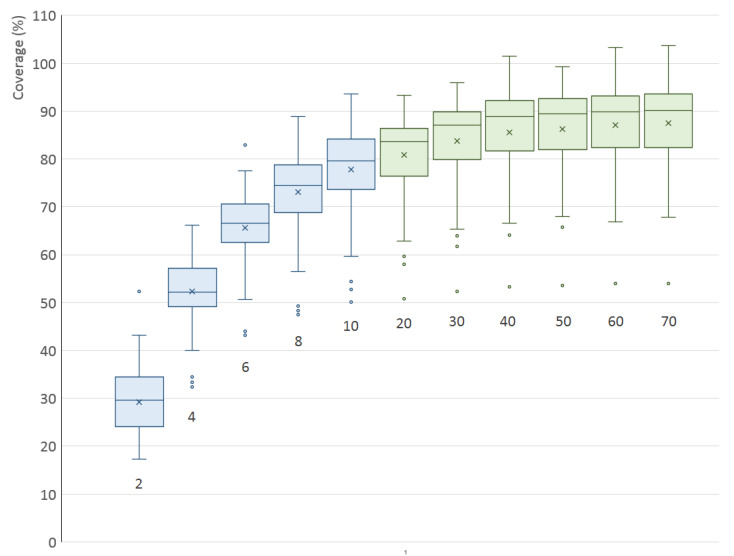
Surface coverage (%) for various number of turntable rotation steps in a two-scanner setup. The colors are used to distinguish different step increments (2, 10).

The results are shown in Figure 15; the averaged, minimal and maximal values are also listed in Table 3. The trends are very clear and consistent—a higher number of steps means better coverage but the increase quickly slows down. When taking the medians into account, there is almost no increase above 60 steps and already at 30 steps the value is at approximately 96% of the maximum.

The best coverage (median) in the previous experiment with 10 steps was around 80% and, as can be seen now, this number can be increased to approximately 90%.

Theoretically, the coverage value should always be lower than 100%, as every object is standing on some surface which therefore cannot be visible to the scanners at all. Nonetheless, some of the coverage values for higher number of rotation steps reach up to almost 104%—this is just a calculation error given by the way the surface coverage is calculated.

The optimal value of rotation steps depends on the particular situation, because higher values create more detailed combined scans (point clouds) but at the cost of more time required for the whole scanning operation (the number of steps directly corresponds to the number of taken individual scans) as well as more time required to process the point cloud (voxelization, etc.). Nevertheless, it can be stated that there is no significant improvement above 30 steps (increment angle 12${}^{\circ }$). If time is an issue, even values between 10 and 20 steps can be acceptable. The coverage drops rapidly for a number of steps less than 10.

### 4.4. Influence of Placement Accuracy

In the previous simulations, the scanned object was placed on the virtual turntable with its center point (the geometric center of the solid body) on the axis of rotation of the turntable (see point *T* on Figure 7). In real scanning, the user of the device would not be able to easily locate the center point for asymmetrical objects and would not place the object precisely. Therefore, the following experiment was executed to find the influence of the object placement accuracy.

This experiment was done for a single scanner with the elevation angle $\delta \in \langle 0^{\circ },70^{\circ }\rangle$. For each elevation angle, there are six different placement offsets of the scanned object—zero offset (the same ideal placement as in the first experiment, see Section 4.1) followed by offsets 10%, 20%, 30%, 40% and 50% relative to the object size. The largest offset from the rotation axis of the turntable is equal to half the size of the scanned object.

**Figure 16:**
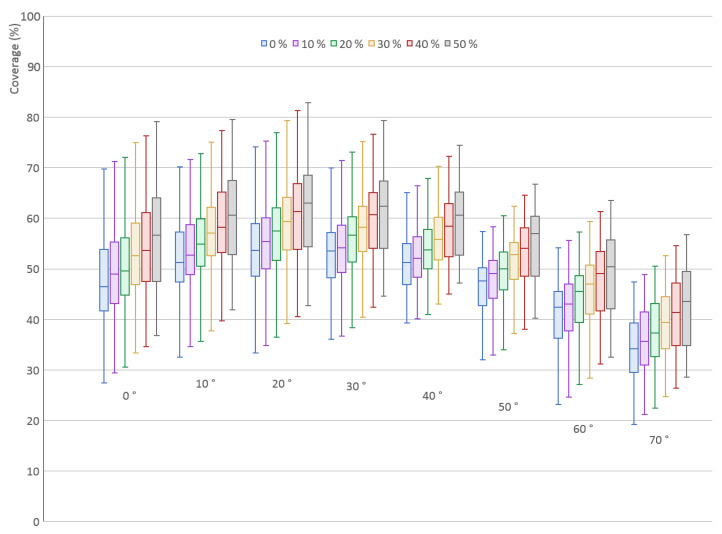
Surface coverage (%) for different elevation angles (°) of a single TOF scanner for various placement offset values (percent of the object size) from the turntable axis of rotation.

The results for all 200 models are shown in Figure 16 in the form of a box plot. It is clear that placing the object further from the center of the turntable actually increases the coverage. This is given by the fact that, during the rotation of an object placed off-center, the scanner can see some surfaces of the object from slightly different angles as the object rotates and moves further and closer to the scanner. The spread increases with the offset because large objects start to move out of view of the scanner. The most important point is that the overall trend stays the same and the optimal angle is still $\delta =20^{\circ }$.

The experiment proved that it is not necessary to place the object precisely to the center of the turntable, and off-center placement may even be advantageous. However, care must be taken for larger objects that could easily get out of view of the scanner. In addition, some algorithms merging the individual scans into one point cloud may not work properly if the placement is too off-center.

## 5. Principal Component Analysis

The simulation experiments described above focused on finding the scanning system configuration that maximizes the surface coverage of the scanned object. However, if just the general shape of the scanned object needs to be captured, for example, to be used in a quick pre-processing step of the point cloud matching algorithm, a method such as the PCA (principal component analysis) can be used to describe the properties of the point cloud.

PCA is a data analysis method commonly used for dimensionality reduction of a data set while preserving the original data variation and distribution in space [30]. The idea of applying PCA on a point cloud is to find an ellipsoid (described by the three semi-axes) that fits the point cloud. The lengths of the ellipsoid semi-axes can be used as a simplified description of the distribution of the points in space.

This is done by first calculating the sample mean $\bar{P}$ and the 3x3 covariance matrix C of the point cloud:

(14) $$\bar{P}=\frac{1}{n}\sum_{i=1}^{n}P_{i},\;C_{j,k}=\frac{1}{n}\sum_{i=1}^{n}{\left(P_{i}-\bar{P}\right)}_{j}{\left(P_{i}-\bar{P}\right)}_{k},$$

where $P_{i}$ is the ith point in the cloud, $i\in \langle 1,n\rangle$. The matrix C is symmetric and positive semi-definite and has three eigenvalues ($\lambda_{1},\lambda_{2},\lambda_{3}$). Together with the corresponding unit eigenvectors ($v_{1},v_{2},v_{3}$), they form the three semi-axes of the ellipsoid

(15) $${\tilde{\lambda }}_{1}\;v_{1},\;{\tilde{\lambda }}_{2}\;v_{2},\;{\tilde{\lambda }}_{3}\;v_{3},$$

where ${\tilde{\lambda }}_{i}=2\sqrt{\lambda_{i}}$ is the length of the semi-axis. If we find the semi-axes for the reference point cloud covering evenly the whole surface (Figure 10) and for the point cloud acquired by scanning, we can evaluate the difference ε by using the standard Euclidean norm

(16) $$\varepsilon =\frac{\left|\tilde{\lambda }-{\tilde{\lambda }}^{\prime }\right|}{\left|\tilde{\lambda }\right|}=\frac{\sqrt{{\left({\tilde{\lambda }}_{1}-{\tilde{\lambda }}_{1}^{\prime }\right)}^{2}+{\left({\tilde{\lambda }}_{2}-{\tilde{\lambda }}_{2}^{\prime }\right)}^{2}+{\left({\tilde{\lambda }}_{3}-{\tilde{\lambda }}_{3}^{\prime }\right)}^{2}}}{\sqrt{{\left({\tilde{\lambda }}_{1}\right)}^{2}+{\left({\tilde{\lambda }}_{2}\right)}^{2}+{\left({\tilde{\lambda }}_{3}\right)}^{2}}},$$

where $\tilde{\lambda }=\left({\tilde{\lambda }}_{1},{\tilde{\lambda }}_{2},{\tilde{\lambda }}_{3}\right)$ and ${\tilde{\lambda }}^{\prime }=\left({\tilde{\lambda }}_{1}^{\prime },{\tilde{\lambda }}_{2}^{\prime },{\tilde{\lambda }}_{3}^{\prime }\right)$ are the semi-axes of the ellipsoids of the scanned point cloud and the reference point cloud, respectively. In an ideal case, ε=0.

As the PCA can be used mainly for a quick initial pre-processing of the point cloud, it is convenient to use as few individual scans as possible—thus also as few rotations of the turntable as possible. Therefore, the focus of the following simulation experiments was to find the minimal required number of individual scans to get a sufficiently accurate ellipsoid semi-axes lengths, or, in other words, a sufficiently small ε (16).

**Figure 17:**
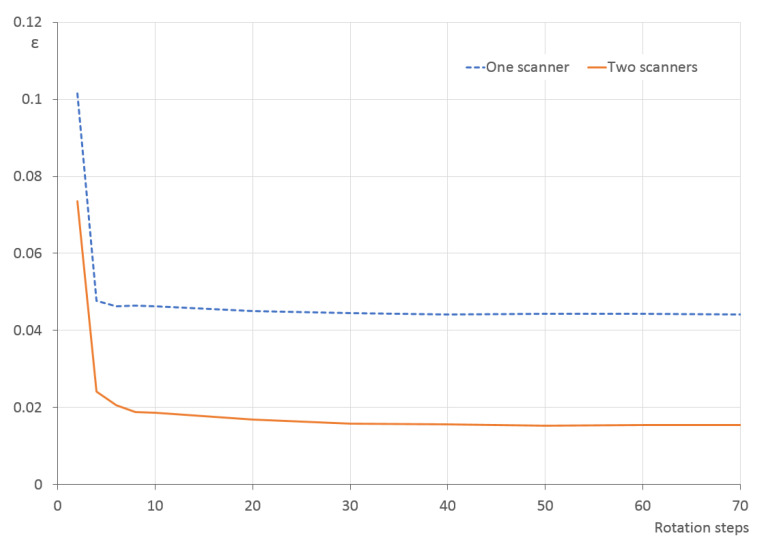
Ellipsoid semi-axes difference ε between the scanned and reference point clouds for various number of turntable rotation steps in the single-scanner and two-scanner setups, averaged for all 200 models.

**Figure 18:**
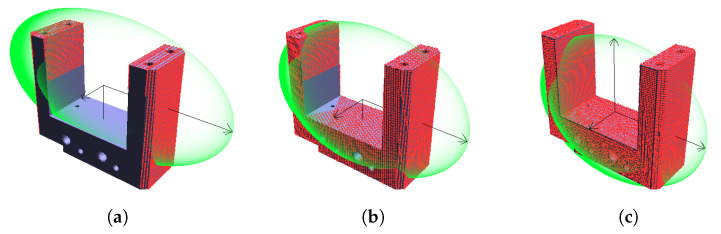
Comparison of point clouds and their ellipsoids: (**a**) one scanner and two turntable rotations; (**b**) one scanner and three turntable rotations; (**c**) reference point cloud.

The experiment was done for the optimal scanner elevations discovered earlier, first for a single scanner ($\delta =20^{\circ }$) and then for two scanners ($\delta_{1}=-20^{\circ },\;\delta_{2}=40^{\circ }$). The number of steps is variable and ranges from 2 to 10 in steps of 2 and then from 20 to 70 in steps of 10. The ε values averaged across all 200 models are shown in Figure 17; Table 4 presents the minimal and maximal values. It is clear that the ε values are much larger for two rotation steps and quickly decrease for three and more steps. The extreme difference for two rotation steps is visible in the example in Figure 18; with just two rotation steps, the point cloud covers only some parts of the object surface and does not capture the overall shape. The situation is much better already for three steps.

Similar to the surface coverage and—as expected—also here the two-scanner setup provides better results. For both cases, it can be stated that the improvement is negligible above 10 rotation steps and virtually zero above 30 steps. Even as few as three or four steps can be sufficient for a quick determination of the ellipsoid properties.

## 6. Physical Experiment

**Figure 19:**
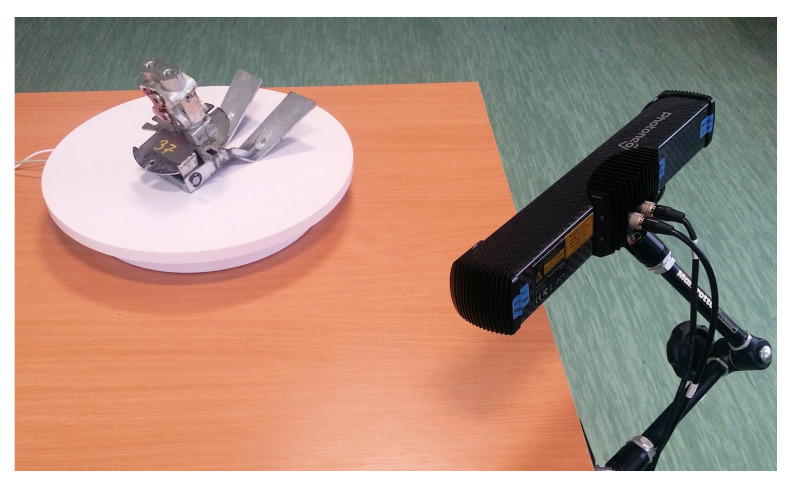
The physical turntable with a Photoneo 3D scanner positioned for a low-angle scanning.

An experiment was performed using a real turntable scanning system PhoXi 3D Meshing consisting of one PhoXi 3D Scanner M, an automatic turntable and a meshing software (all provided by Photoneo). The simple setup is shown in Figure 19. The scanner can be manually positioned to the required elevations above the turntable; the angle was measured using a digital inclinometer.

**Figure 20:**
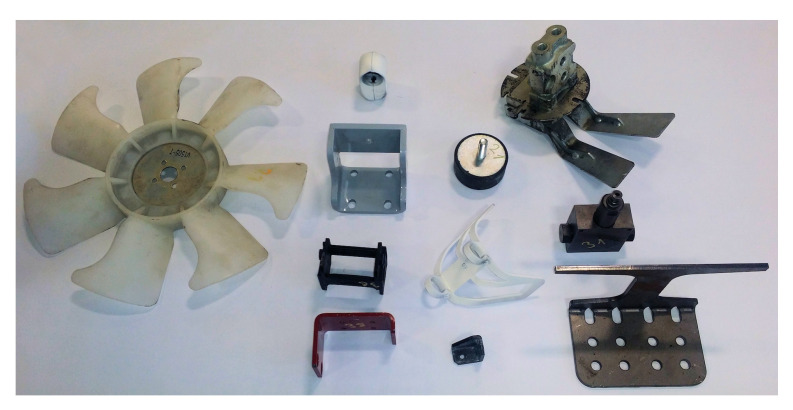
A subset of the mechanical parts and sub-assemblies used in the physical experiment.

The aim of this experiment was to verify whether the results from simulated scanning correspond to the results from a real scanner. The idea was to perform the first experiment (finding the optimal elevation of a single scanner) on the real system using a subset of the set of 200 models. It is not possible to use the whole set because most of the manufactured parts are not available and the experiment would also take a very long time. The subset consists of 15 objects—most of the real parts are shown in Figure 20. The corresponding 3D models are presented above in Figure 8.

The physical scanner used in this experiment uses triangulation with structured light in the visible spectrum, the distance between the emitter and detector is *L* = 350 mm, the horizontal FOV of the emitter is $47^{\circ }$ and the vertical FOV is $40^{\circ }$. These parameters were also used in the simulation for this experiment.

### 6.1. Evaluation Criterion

**Figure 21:**
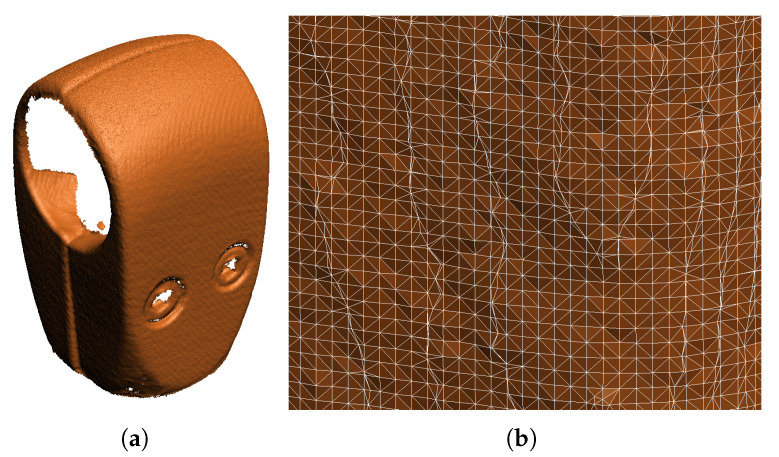
A sample output from the real 3D scanner: (**a**) the overall view of the mesh; (**b**) a detail of the vertex grid of the generated triangle mesh.

The main criterion is the same as for simulated scanning, i.e., the percentage of the part surface covered by the point cloud. However, the implementation of this criterion is different. The PhoXi 3D Meshing software returns the scanned model in the form of a triangle mesh with vertices arranged in a semi-uniform grid (see Figure 21) with the cell size of 0.1526×0.1526 mm. Even on a scan of a flat surface, the grid is not perfectly flat because of noise. Thus, it would be inaccurate to calculate the area of all the small triangles and add them together to get the scanned area of the object. Instead, we simply calculate the number of the vertices *n* and use the known grid cell size to get the equivalent total surface area

(17) $$A=0.1526^{2}\cdot n$$

The reference area is calculated from the original STL model as a sum of the areas of all triangles using the Heron’s formula

(18) $$A_{ref}=\frac{1}{4}\sum_{i=0}^{t}\sqrt{\left(a_{i}+b_{i}+c_{i}\right)\left(-a_{i}+b_{i}+c_{i}\right)\left(a_{i}-b_{i}+c_{i}\right)\left(a_{i}+b_{i}-c_{i}\right)},$$

where *t* is the number of triangles in the STL mesh and $a_{i}$, $b_{i}$ and $c_{i}$ are the lengths of the three sides of the *i*th mesh triangle. The surface coverage is then calculated as $\frac{A}{A_{ref}}$.

The PCA criterion was also used for the real experiment, to verify how much the overall spacial distribution of points in the scanned point cloud corresponds to the original object. In this case, it can be of a bigger importance because the surface coverage criterion is not able to distinguish in the scanned point cloud points that represent some real object surfaces and points that are erroneous data (noise, reflections, etc.). While these surplus points always misleadingly improve the surface coverage criterion, the ellipsoid semi-axes can be affected negatively by them—which is the proper behavior.

### 6.2. Results and Discussion

As mentioned above, this experiment tried to find the optimal elevation angle for a single triangulation scanner. The number of rotation steps of the turntable is constant and equals 10 (increment angle 36°); the elevation angle is $\delta \in \langle 10^{\circ },70^{\circ }\rangle$ with $10^{\circ }$ increments. Note that it was not possible to use $\delta <10^{\circ }$ because the real scanner performs a calibration with the turntable and must be able to see a calibration pattern placed on the table. Other types of 3D scanners may not have this requirement (or some other way of calibration may be used instead), so even lower angles may be possible.

**Figure 22:**
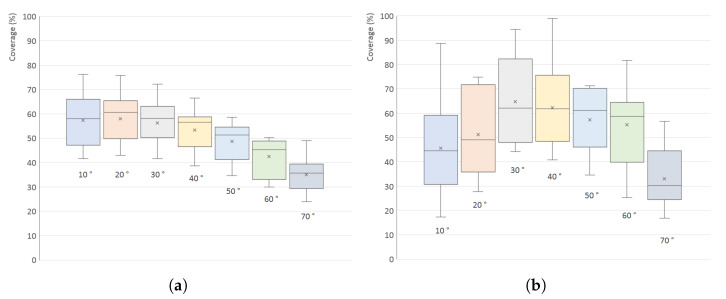
Surface coverage (%) for different elevation angles (°) of a single triangulation scanner for selected 15 models: (**a**) results from simulated scanning (triangulation scanner); (**b**) results from the real 3D scanner.

The surface coverage results for 15 models are shown in Figure 22 in the form of a box plot; the image contains both the graph from the simulated scanning and from the real scanning to provide a quick comparison. As can be seen, the values from real scanner have a much greater spread which is given by a large amount of noise in the data. The comparison shows similarities for elevation angles above 30° lower elevations have smaller surface coverage in real scanning.

**Figure 23:**
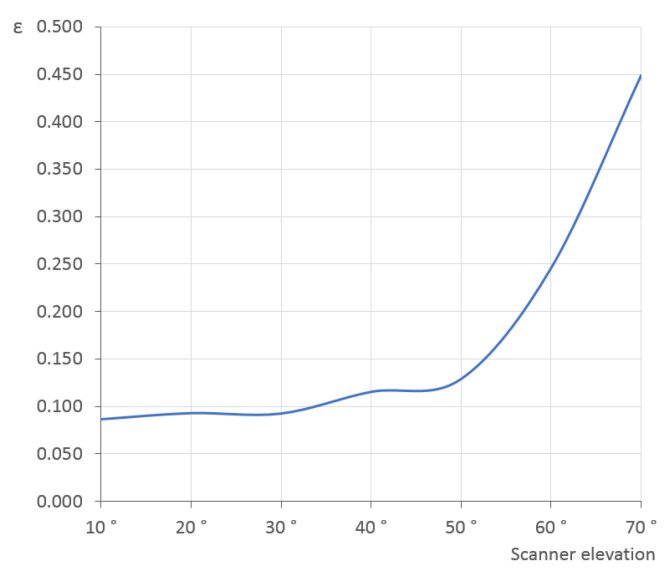
Ellipsoid semi-axes difference ε between the scanned (by the real 3D scanner) and reference point clouds for various elevation angles (°) of a single scanner, averaged for the selected 15 models.

The PCA results are shown in Figure 23. The lower elevation angles ($\delta \leq 30^{\circ }$) provide better results (lower ε values), which is explained in the following section.

### 6.3. Compensating for Reflection Errors

**Figure 24:**
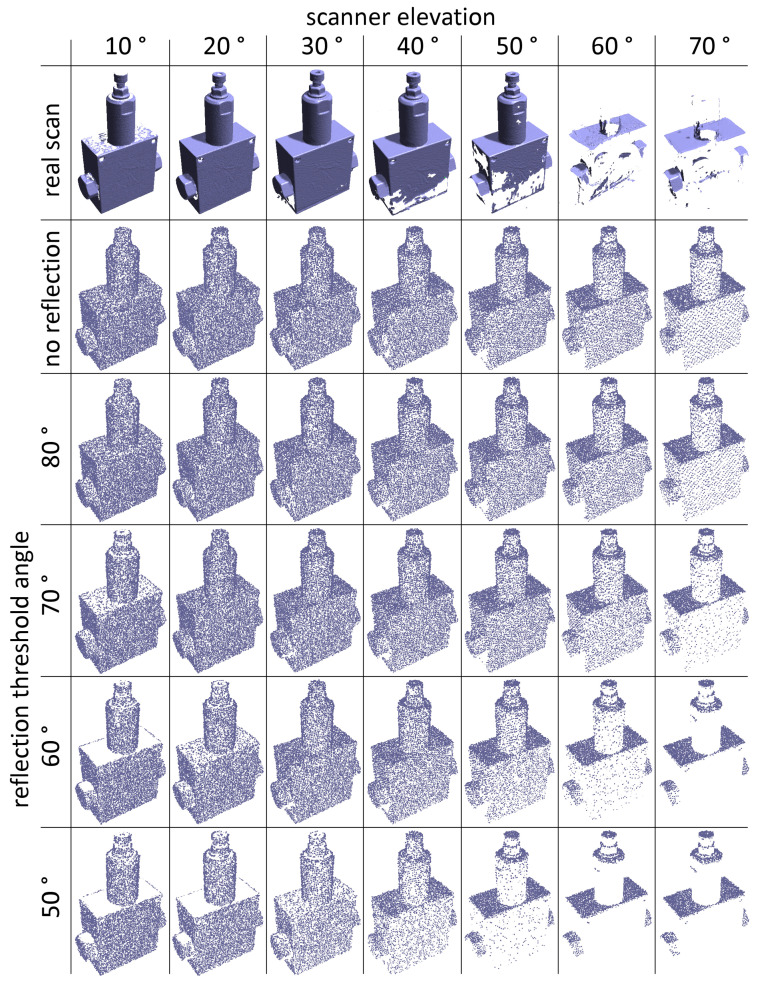
Real (the top-most line) and simulated merged scans of the same mechanical part made by one scanner at various elevation angles (horizontal axis) and various threshold angles α for simulated reflection errors (vertical axis).

The topmost row in Figure 24 shows an example of a mechanical part with the common behavior during scanning—the horizontal surfaces get smaller coverage percentage for lower scanner elevation angles and the vertical surfaces get smaller coverage percentage for higher scanner elevation angles. In this particular case of a metallic object, this is even worse for the real scanning with elevation angles above 50°, because surface reflectivity further reduces the coverage and the PhoXi 3D Meshing software removes sparse areas during filtering and mesh building.

**Figure 25:**
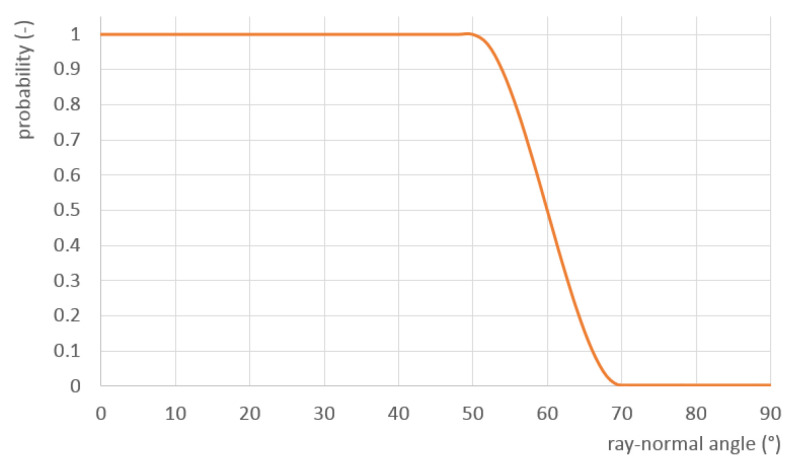
Approximation of reflectivity errors—probability of a scanned point being retained in the point cloud based on the angle between the laser ray and the surface normal (threshold angle α = 60°, spread angle σ = 10°).

The real impact of surface reflectivity on the quality of a 3D scan is a complex phenomenon [41]. To try to emulate this behavior in the simulation, the following simple approximation of reflection errors was added—the angle between the laser ray and and the surface normal vector is calculated, and, if this angle is above a certain threshold, the point gets a random chance of being discarded. The probability that a particular point remains in the final point cloud is calculated as (see also Figure 25)

(19) $$P=\left\{\begin{array}{cc} 1, & \mathrm{if}\;\gamma \leq \alpha -\sigma \\ 2\gamma_{r}^{3}-3\gamma_{r}^{2}+1, & \mathrm{if}\;\alpha -\sigma <\gamma <\alpha +\sigma \\ 0, & \mathrm{if}\;\gamma \geq \alpha +\sigma , \end{array}\right.$$

where $\vec{n}$ is the surface normal vector, $\vec{d}$ is the laser ray vector, $\gamma =\arccos(-\vec{d}\cdot \vec{n})$ is the angle between $\vec{n}$ and $\vec{d}$, α is the threshold angle, σ is the spread angle and $\gamma_{r}$ is a relative angle

(20) $$\gamma_{r}=\frac{\gamma -\left(\alpha -\sigma \right)}{2\sigma }.$$

**Figure 26:**
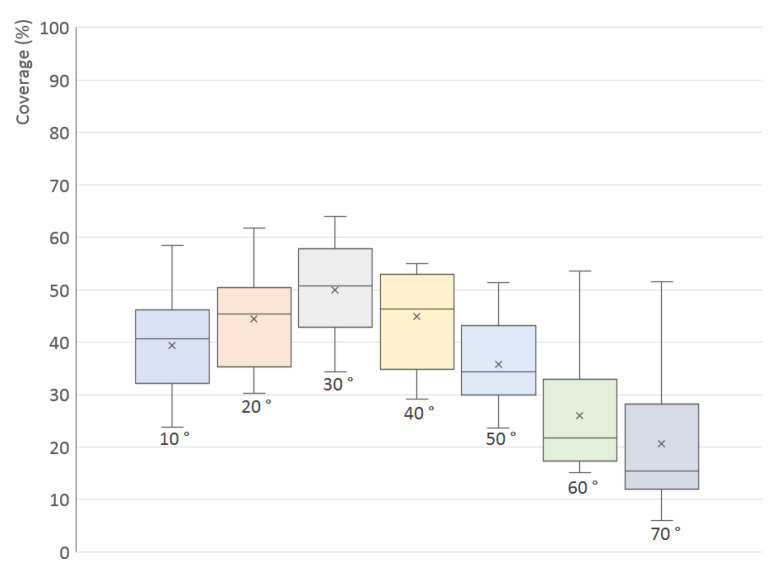
Surface coverage (%) for different elevation angles (°) of a single simulated triangulation scanner for 15 selected models with approximate simulation of reflection errors (α = 60°, σ = 10°).

Figure 24 shows that the simulation results are visually closest to the real scans (the top-most row) on the penultimate row, i.e., when the threshold angle is α = 60° (the spread angle was σ = 10°). The top base of the cylindrical part of the object is missing on the real scan because it appears too disjoint with the rest of the model and the meshing software removes it as a noise. With this setting, the results from the virtual scanning of all 15 models changes from the ones in Figure 22a to those in Figure 26. These results are slightly closer to the actual values from the real scanner (Figure 22b), as far as the overall trend is concerned.

## 7. Conclusions

The research concerns a particular use case where a scanning device is used to automatically recognize a mechanical part by comparing the merged point cloud acquired by scanning with a database of parts. The virtual scanning application was created not only to find the optimal configuration of the physical scanning device but also to get a large amount of testing data for tuning and debugging the recognition algorithm before the physical scanner is built.

The evaluation criterion of the optimal scanning device configuration is the achievable coverage of the object surface because information about as many details of the shape as possible is required to be able to properly distinguish the 3D model. The scanning device will consist of a rotating table and one or two physical scanners.

A single scanner together with a turntable cannot provide sufficient coverage of surfaces of common mechanical parts—the coverage typically stays around 60%, even for the optimal scanner elevation $\delta =20^{\circ }$. This can be expected because non-symmetrical objects must be scanned not only from multiple horizontal angles, but also from different elevations. Using two scanners with the discovered optimal elevations $\delta_{1}=-20^{\circ }$ and $\delta_{2}=40^{\circ }$ and 10 rotation steps of the turntable allows scanning on average almost 80% of the surface, which may be acceptable in most cases. By increasing rotation steps to 30, the coverage reaches almost 90%.

When just the general distribution of mass of the scanned object is concerned, for example during a pre-selection step that limits the required number of detailed comparisons of the scanned point cloud with the database of parts, PCA can be used to get the lengths of the ellipsoid semi-axes. For this optional step, the above-mentioned scanner elevation angles can be used and the number of turntable rotation steps for this purpose can be even smaller than the recommended number for high surface coverage, even down to three or four, while preserving an acceptable small error when compared to the ideal ellipsoid of the reference point cloud. Using PCA to calculate the ellipsoid semi-axes lengths can also be used to detect some erroneous data that misleadingly improve surface coverage by creating areas in the point cloud that do not correspond to a real surface, for example due to reflection.

The user of the scanning device is responsible for choosing the placement orientation of the object on the turntable—the interesting features and details should be oriented upwards and the object should be placed in the middle of the table (the centering does not have to be precise; slightly off-center positions may even improve the results). There are, however, models that will always have some important details obstructed by the turntable plate regardless the chosen orientation. To be able to properly and reliably distinguish these objects, it may be necessary to turn the object over after scanning, perform additional scans of the bottom part, and then merge the point clouds together. It is also important to mention that the recommended configuration is a compromise that should provide on average the best possible results for a huge variety of shapes, but it can be a bad solution for some specific shapes. If the particular application focuses mostly on models with similar properties, these models should be used to find the optimal configuration.

Better coverage can also be achieved by making scans from more than two elevations. This is possible even with a single physical scanner by making a mechanism that automatically changes the elevation of the scanner after each complete turntable revolution.

An experiment on a real scanning turntable with a triangulation scanner confirmed the general trend of surface coverage at various elevation angles, but it also showed some dissimilarities, which were further reduced by implementing a rough approximation of errors caused by surface reflectivity. It would require a larger set of samples to find the real optimal turntable configurations. Nonetheless, the configurations discovered based on the results of simulations with 200 samples would probably be the best candidates to try.

The experiment showed that the material properties of scanned objects and the optical properties of the real scanning device can alter the results. The simulation results can be considered reasonably accurate and valid as far as geometric properties are concerned, especially when at least a rough approximation of reflectance is applied. Nevertheless, future work may include more accurate simulation of measurement errors and noise in the data, with a focus especially on the reflectance of the scanned object, which is one of the main sources of error for optical scanners. The matter of influence of material properties on the scanning results was already partially addressed by some of the authors of this paper (see [42] for more details).

## Figures and Tables

**Table 1:** Minimal, averaged and maximal surface coverage (%) for different elevation angles δ of a single TOF scanner.

Elevation δ	Minimum	Mean	Median	Maximum
0${}^{\circ }$	27.4%	47.2%	46.4%	73.2%
10${}^{\circ }$	30.5%	51.5%	51.3%	74.6%
20${}^{\circ }$	30.7%	52.9%	53.6%	74.1%
30${}^{\circ }$	29.4%	52.3%	53.5%	70.6%
40${}^{\circ }$	28.0%	49.8%	51.2%	67.5%
50${}^{\circ }$	25.4%	45.6%	47.6%	62.1%
60${}^{\circ }$	22.3%	40.6%	42.3%	54.1%
70${}^{\circ }$	19.1%	34.0%	34.1%	47.4%

**Table 2:** Minimal, averaged and maximal surface coverage (%) for various combinations of elevation angles $\delta_{1}$ and $\delta_{2}$ of two scanners.

Elevation $\delta_{1}$	Elevation $\delta_{2}$	Minimum	Mean	Median	Maximum
−20${}^{\circ }$	10${}^{\circ }$	46.5 %	72.9 %	73.4 %	93.6 %
−20${}^{\circ }$	20${}^{\circ }$	48.2 %	75.8 %	77.0 %	94.4 %
−20${}^{\circ }$	30${}^{\circ }$	48.8 %	76.9 %	78.4 %	94.4 %
−20${}^{\circ }$	40${}^{\circ }$	49.1 %	77.7 %	79.8 %	94.0 %
−20${}^{\circ }$	50${}^{\circ }$	48.8 %	77.3 %	79.5 %	95.1 %
−20${}^{\circ }$	60${}^{\circ }$	48.4 %	76.4 %	78.2 %	95.7 %
−20${}^{\circ }$	70${}^{\circ }$	47.7 %	74.9 %	77.2 %	96.8 %
−10${}^{\circ }$	20${}^{\circ }$	43.6 %	67.4 %	67.3 %	91.6 %
−10${}^{\circ }$	30${}^{\circ }$	45.4 %	69.0 %	68.7 %	92.2 %
−10${}^{\circ }$	40${}^{\circ }$	46.2 %	69.6 %	69.3 %	91.8 %
−10${}^{\circ }$	50${}^{\circ }$	43.6 %	69.3 %	69.0 %	90.1 %
−10${}^{\circ }$	60${}^{\circ }$	42.9 %	68.8 %	68.8 %	88.7 %
−10${}^{\circ }$	70${}^{\circ }$	41.3 %	67.5 %	67.1 %	89.4 %
0${}^{\circ }$	30${}^{\circ }$	40.0 %	64.6 %	64.8 %	88.9 %
0${}^{\circ }$	40${}^{\circ }$	40.5 %	64.7 %	64.3 %	88.3 %
0${}^{\circ }$	50${}^{\circ }$	39.4 %	64.1 %	64.3 %	86.9 %
0${}^{\circ }$	60${}^{\circ }$	38.0 %	63.1 %	63.6 %	84.9 %
0${}^{\circ }$	70${}^{\circ }$	36.5 %	61.8 %	62.5 %	82.1 %
10${}^{\circ }$	40${}^{\circ }$	39.1 %	64.5 %	65.7 %	88.3 %
10${}^{\circ }$	50${}^{\circ }$	38.8 %	63.7 %	64.9 %	86.9 %
10${}^{\circ }$	60${}^{\circ }$	37.8 %	62.7 %	64.0 %	84.9 %
10${}^{\circ }$	70${}^{\circ }$	36.5 %	61.3 %	62.5 %	82.5 %
20${}^{\circ }$	50${}^{\circ }$	37.4 %	63.0 %	64.0 %	86.1 %
20${}^{\circ }$	60${}^{\circ }$	36.1 %	61.9 %	63.4 %	83.6 %
20${}^{\circ }$	70${}^{\circ }$	35.3 %	60.6 %	62.3 %	81.6 %
30${}^{\circ }$	60${}^{\circ }$	34.2 %	61.2 %	62.4 %	82.0 %
30${}^{\circ }$	70${}^{\circ }$	33.0 %	59.7 %	61.2 %	80.8 %
40${}^{\circ }$	70${}^{\circ }$	31.2 %	56.8 %	59.3 %	75.0 %

**Table 3:** Minimal, averaged and maximal surface coverage (%) for various number of rotation steps of the turntable, using two scanners with optimal elevations.

Steps	Minimum	Mean	Median	Maximum
2	17.2%	29.2%	29.6%	52.3%
4	32.4%	52.4%	52.2%	66.2%
6	43.1%	65.7%	66.6%	82.9%
8	47.4%	73.1%	74.5%	88.8%
10	50.1%	77.8%	79.7%	93.6%
20	50.8%	80.8%	83.7%	93.3%
30	52.3%	83.7%	87.1%	95.9%
40	53.3%	85.6%	88.9%	101.5%
50	53.5%	86.2%	89.5%	99.2%
60	53.9%	87.1%	89.8%	103.3%
70	54.0%	87.5%	90.1%	103.6%

**Table 4:** Minimal, averaged and maximal ellipsoid differences ε for various number of rotation steps of the turntable, using one (^1^) and two (^2^) scanners with optimal elevations.

Steps	Min ^1^	Mean ^1^	Max ^1^	Min ^2^	Mean ^2^	Max ^2^
2	0.031	0.102	0.201	0.016	0.074	0.156
4	0.006	0.048	0.095	0.002	0.024	0.071
6	0.007	0.046	0.090	0.004	0.021	0.068
8	0.008	0.046	0.088	0.003	0.019	0.063
10	0.006	0.046	0.088	0.003	0.019	0.066
20	0.008	0.045	0.087	0.001	0.017	0.068
30	0.009	0.044	0.087	0.002	0.016	0.068
40	0.009	0.044	0.088	0.001	0.016	0.068
50	0.009	0.044	0.089	0.000	0.015	0.068
60	0.010	0.044	0.089	0.001	0.015	0.069
70	0.010	0.044	0.088	0.002	0.015	0.068

## Data Availability

The data presented in this study are available on request from the corresponding author. The data are not publicly available due to funding project restrictions.

## Abbreviations

The following abbreviations are used in this manuscript:

CAD	Computer-aided design
GPU	Graphics processing unit
LADAR	Laser detection and ranging
PCA	Principal component analysis
ROS	Robot operating system
TOF	Time of flight
STL	Stereolithography
